# Nbn and Atm Cooperate in a Tissue and Developmental Stage-Specific Manner to Prevent Double Strand Breaks and Apoptosis in Developing Brain and Eye

**DOI:** 10.1371/journal.pone.0069209

**Published:** 2013-07-30

**Authors:** Paulo M. G. Rodrigues, Paulius Grigaravicius, Martina Remus, Gabriel R. Cavalheiro, Anielle L. Gomes, Mauricio R. Martins, Lucien Frappart, David Reuss, Peter J. McKinnon, Andreas von Deimling, Rodrigo A. P. Martins, Pierre-Olivier Frappart

**Affiliations:** 1 Clinical Cooperation Unit Neuropathology, German Cancer Research Center (DKFZ), Heidelberg, Germany; 2 Programa de Biologia Celular e do Desenvolvimento, Instituto de Ciências Biomédicas, Universidade Federal do Rio de Janeiro, Rio de Janeiro, Brazil; 3 Programa de Pós Graduação em Biofísica, IBCCF, Universidade Federal do Rio de Janeiro, CCS, Rio de Janeiro, Brazil; 4 Department of Neuropathology, Institute of Pathology, Ruprecht-Karls-Universität Heidelberg, Heidelberg, Germany; 5 Leibniz Institute for Age Research – Fritz Lipmann Institute (FLI), Jena, Germany; 6 Department of Genetics, St.Jude Children's Research Hospital, Memphis, Tennessee, United States of America; University Medical Center Hamburg-Eppendorf, Germany

## Abstract

Nibrin (NBN or NBS1) and ATM are key factors for DNA Double Strand Break (DSB) signaling and repair. Mutations in *NBN* or *ATM* result in Nijmegen Breakage Syndrome and Ataxia telangiectasia. These syndromes share common features such as radiosensitivity, neurological developmental defects and cancer predisposition. However, the functional synergy of Nbn and Atm in different tissues and developmental stages is not yet understood. Here, we show *in vivo* consequences of conditional inactivation of both genes in neural stem/progenitor cells using *Nestin-Cre* mice. Genetic inactivation of *Atm* in the central nervous system of Nbn-deficient mice led to reduced life span and increased DSBs, resulting in increased apoptosis during neural development. Surprisingly, the increase of DSBs and apoptosis was found only in few tissues including cerebellum, ganglionic eminences and lens. In sharp contrast, we showed that apoptosis associated with *Nbn* deletion was prevented by simultaneous inactivation of *Atm* in developing retina. Therefore, we propose that Nbn and Atm collaborate to prevent DSB accumulation and apoptosis during development in a tissue- and developmental stage-specific manner.

## Introduction

The central nervous system (CNS) exhibits an acute sensitivity to double-strand breaks (DSBs) during its development [1]. Indeed, the majority of the human diseases associated with mutations in DSB signaling or repair genes present a wide spectrum of neurological abnormalities ranging from microcephaly to neurodegeneration [1].

Hypomorphic mutations in NBN lead to the Nijmegen Breakage Syndrome (NBS, OMIM 251260) a rare autosomal recessive disorder associated with growth retardation, immunodeficiency, neurological defects, radiosensitivity and tumor predisposition, including astrocytomas and medulloblastomas. Notably, CNS malignancies are rarely found in NBS and other related inherited diseases, such as Ataxia-Telangiectasia (A-T, ATM) [2], [3]. In fact, these common features are expected, since the nibrin (NBN) protein is a target of DNA damage signaling kinases, such as ATM or ATR, and is a component of the MRN complex (with RAD50 and MRE11) that is involved in DNA damage signaling and repair, telomere maintenance, cell cycle checkpoint activation and processing of stalled replication forks [4]. NBN is a key sensor of the DSBs and is essential for the efficient activation of DNA repair PI-3 like kinases ATM or ATR in response to both exogenous and endogenous DNA damaging agents such as ionizing radiation (IR), ultra-violet (UV) and stalled replication forks [5]. Phosphorylation of NBN at serines 278 and 343 by the same PI-3 kinases is required for the activation of the intra-S phase checkpoint [6]–[8]. Finally, Nbn has also been shown to be required for the DSB repair branching between the Non Homologous End Joining (NHEJ) and Homologous Recombination Repair (HRR) [9].

The inactivation of *Nbn* leads to early embryonic lethality, while the hypomorphic mutant mice are viable and barely exhibit the NBS-associated neurological defects [10]–[14]. The specific inactivation of *Nbn* in mouse neural tissues using *Nestin-cre* transgenic mice results in a combination of the neurological abnormalities of NBS, A-T and A-TLD, including microcephaly, growth retardation, cerebellar defects and ataxia [15]. Analysis of *Nbn* conditional knockout mice indicated that the loss of Nbn impairs the proliferation of granule cell progenitors and increased apoptosis of post mitotic neurons in the cerebellum [15]. It was also shown that *Nbn* inactivation leads to defects in myelin formation, oligodendrocyte development and astrocyte dysfunction [16]–[18]. In addition, Nbn-deficient neural stem cells exhibit proliferation defects, but not increased apoptosis, and contain more chromosomal breaks accompanied by Atm-mediated p53 activation [15]. Importantly, depletion of p53 significantly rescues the neurological defects of Nbn mutant mice, while inactivation of *Atm* in Nbn-deficient neural stem cells seems to worsen the cerebellar defects of Nbn deficient mice [17]. Apart from neurological defects these mice also exhibit severe eye phenotypes, such as micropthalamia, disorganization of the lens, impaired visual function and cataracts [16], [19].

Even though it is clear that functional interaction between NBN and ATM is required for a proper DNA damage response and that both are crucial for CNS development, it remains unclear whether the functional relationship between NBN and ATM is identical or equally relevant in all developing tissues. For example, nothing or little is known about their functional relationship during eye and brain development. To study how Nbn and Atm are functionally interconnected in the development of these tissues and to better understand the origins of the developmental defects caused by Nbn and/or Atm-deficiency, we simultaneously inactivated them in various neural and eye tissues using multiple Cre/LoxP systems. We report that *Atm* inactivation worsens the Nbn-deficient phenotype causing increased genomic instability and increased apoptosis of neural progenitors. Similar results were observed for progenitor cells of the lens anterior epithelia, but not for retinal progenitor cells (RPC). Interestingly, even though both Nbn and Atm are ubiquitously expressed in the CNS and in the eye [20], [21], we show that the phenotype severity caused by simultaneous inactivation of *Nbn* and *Atm* is restricted to some tissues or to specific stages of development. Therefore, we propose that functional synergy between Nbn and Atm to prevent and repair DSBs and apoptosis during development is highly dependent on the tissue type and its developmental stage.

## Results

### Atm deficiency promotes premature growth defect and death of Nbn^Nes-Cre^ mice

**Figure 1:**
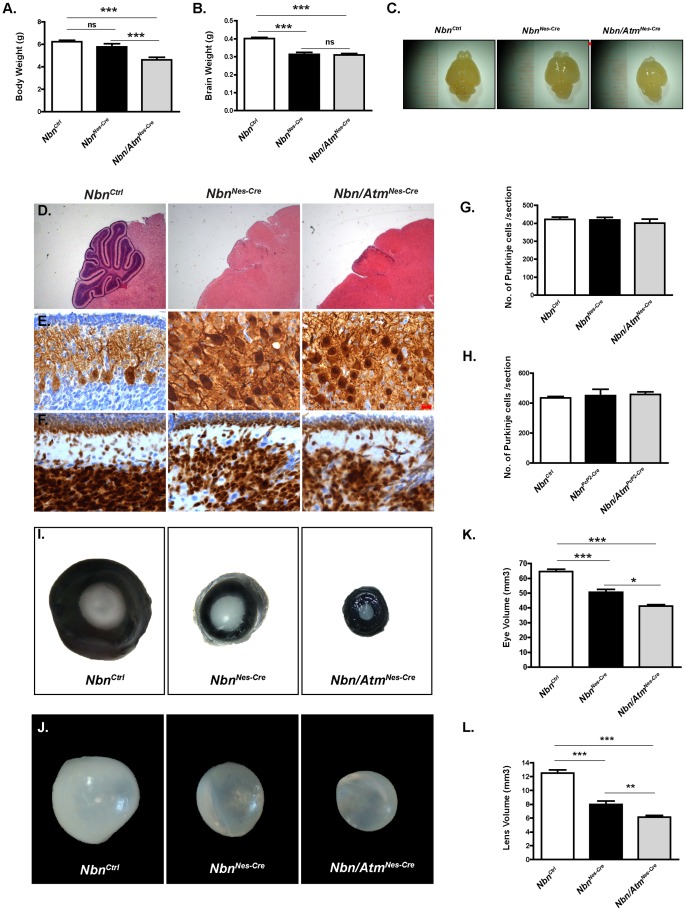
Heterogeneous effects of *Atm* inactivation on Nbn-deficient brain and eye. (**A**) Body weight of P9 *Nbn/Atm^F/F^;Nestin-cre* (*Nbn/Atm^Nes-Cre^*) mice was reduced as compared to control (*Nbn^Ctrl^*) and *Nbn^F/F^;Nestin-Cre* (*Nbn^Nes-Cre^*) littermates. (**B**–**C**) Brain weight (B) and size (C) of P9 of *Nbn^Nes-Cre^* (n = 11) and *Nbn/Atm^Nes-Cre^* (n = 22) mice are significantly reduced as compared to those of *Nbn^Ctrl^* (n = 123), without dramatic disruption of the general morphology. (**D**) H&E staining of *Nbn^Ctrl^*, *Nbn^Nes-Cre^* and *Nbn/Atm^Nes-Cre^* brain sections at P9 reveal reduction of the size of the *Nbn^Nes-Cre^* and *Nbn/Atm^Nes-Cre^* mice cerebella compared to those of *Nbn^Ctrl^* mice (Magnification ×10). (**E**) Calbindin (D-28K) staining shows disruption of the Purkinje cells layer in both *Nbn^Nes-Cre^* and *Nbn/Atm^Nes-Cre^* cerebella (Magnification ×400). (**F**) NeuN staining shows the disorganization of the inner granular layer (IGL) and the dramatic size reduction of the external granular layer (EGL) in *Nbn^Nes-Cre^* and *Nbn/Atm^Nes-Cre^* cerebella (Magnification ×400). (**G**) Purkinje cells number is unchanged in both *Nbn^Nes-Cre^* (n = 5) and *Nbn/Atm^Nes-Cre^* (n = 3) cerebella compared to *Nbn^Ctrl^* (n = 5) cerebella. (**H**) Inactivation of *Nbn* (*Nbn^PcP2-Cre^*; n = 2) or both *Nbn* and *Atm* (*Nbn/Atm^PcP2-Cre^*; n = 2) in post-mitotic Purkinje cells using PcP2-Cre transgene does not alter the number of Purkinje cells in adult cerebellum. (**I**) Genetic inactivation of *Nbn* and *Atm* in the lens epithelia severely impairs eye and lens development. Representative pictures of P9 eyes from *Nbn^Ctrl^*, *Nbn^Nes-Cre^* and *Nbn/Atm^Nes-Cre^* mice. (**J**) Representative pictures of P9 dissected lenses from *Nbn^Ctrl^*, *Nbn^Nes-Cre^* and *Nbn/Atm^Nes-Cre^*. (**K**) Reduction in volume of the Nbn-deficient eyes was potentiated by simultaneous inactivation of both *Nbn* and *Atm*. *Nbn^Ctrl^* (n = 17), *Nbn^Nes-Cre^* (n = 14) and *Nbn/Atm^Nes-Cre^* (n = 28). (**L**) Lens volume measurements show that simultaneous inactivation of *Nbn* and *Atm* results in a greater reduction of lens volume as compared to control or Nbn-deficient lens, *Nbn^Ctrl^*(n = 7), *Nbn^Nes-Cre^* (n = 6) and *Nbn/Atm^Nes-Cre^* (n = 15). Error bars indicate SEM. (* p<0,05, ** p<0,01, *** p<0,001, ns: non-significant).

It has been shown that inactivation of *Nbn* in mouse neural stem cells (*Nbn^Nes-Cre^*) was associated with severe growth defects and premature death around 21 days after birth (P21) [15]. Concomitant deletion of *Atm* and *Nbn* in neural progenitor cells (*Nbn/Atm^Nes-Cre^*) exacerbated this phenotype, albeit *Nbn^Nes-Cre^* and *Nbn/Atm^Nes-Cre^* mice were viable and appeared indistinguishable from their littermates at birth. *Nbn/Atm^Nes-Cre^* exhibited a stronger growth defect at P9, as compared to *Nbn^Nes-Cre^* mice (Figure 1A) and prematurely died by P14. Nevertheless, both genotypes exhibited a similar tremor and ataxia. The brain weight and size were indistinguishable between *Nbn^Nes-Cre^* and *Nbn/Atm^Nes-Cre^* mice at P9 (Figure1 B–C).

### Nbn and Atm are not essential for Purkinje Cells homeostasis

Microscopic examination of the *Nbn^Nes-Cre^* and *Nbn/Atm^Nes-Cre^* P9 brains showed identical granule cell loss, cerebellar and cortical developmental defects by P9 (Figure 1D–F). Although both *Nbn^Nes-Cre^* and *Nbn/Atm^Nes-Cre^* Purkinje cells presented impaired localization and altered dendritic arborization, their numbers were not different from the control mice (Figure 1E, 1G). To test whether Nbn and/or Atm play a role in the homeostasis of differentiated Purkinje cells, *Nbn^PcP2-Cre^* and *Nbn/Atm^PcP2-Cre^* mice were generated. PcP2-Cre/L7-Cre allows a specific genetic inactivation in post-mitotic Purkinje cells during postnatal development (7 days of age/P7) [22]. Macroscopic analysis of *Nbn/Atm^PcP2-Cre^* P21 brains did not reveal abnormalities of the cerebellum structure (data not shown). Histological analysis did not show loss of granule cells, morphological abnormalities or alterations in the number of Purkinje cells (Figure 1H), suggesting that both Nbn and Atm are dispensable for Purkinje cells homeostasis in mouse.

### Atm inactivation does not worsen the Nbn^Nes-Cre^ phenotype in neural stem/progenitor cells

**Figure 2:**
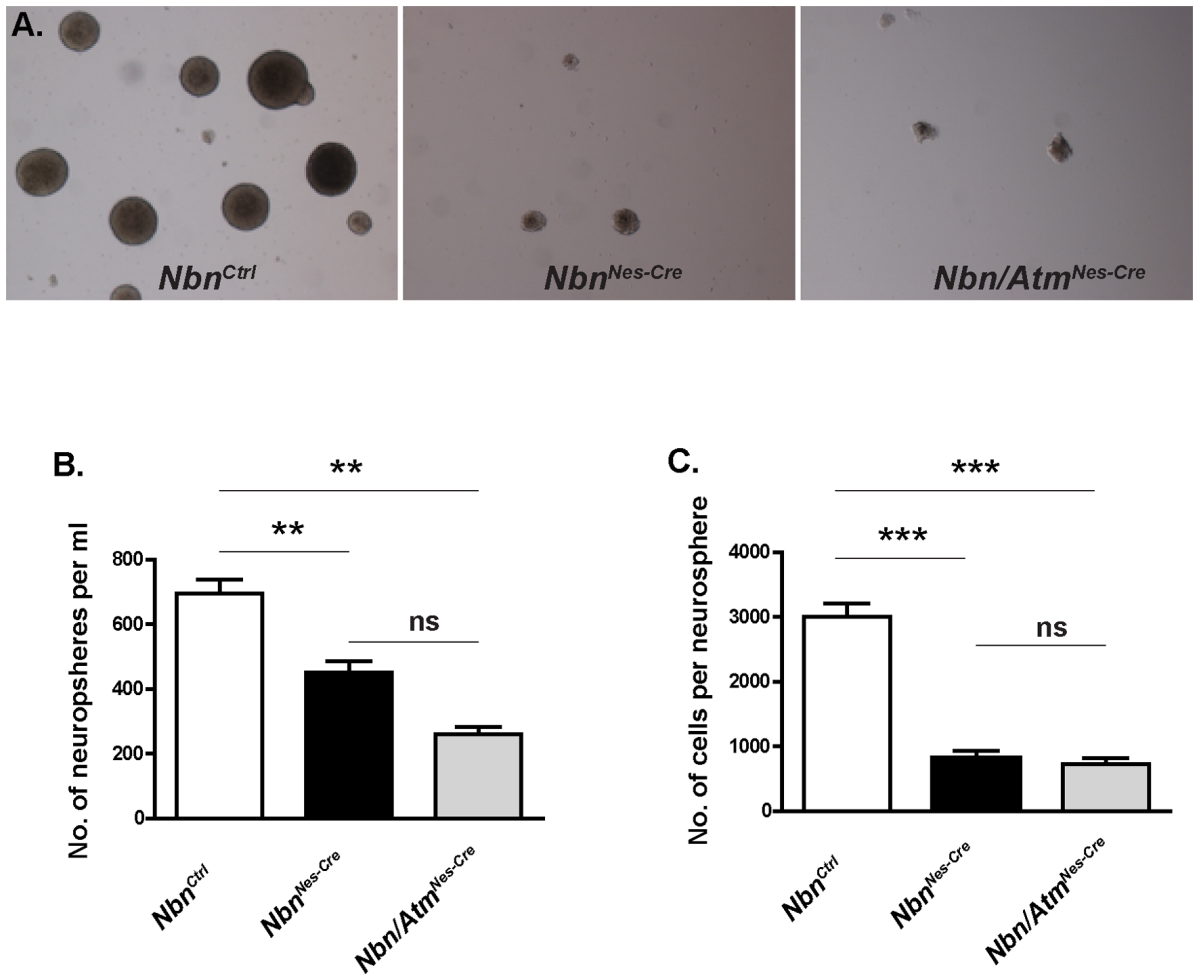
*Nbn^Nes-Cre^* and *Nbn/Atm^Nes-Cre^* neural progenitor cells exhibit identical growth defects. (**A**) Representative pictures of 7 days neurosphere cultures of control, Nbn-deficient and Nbn/Atm-deficient brains (Magnification ×40). (**B**) Numbers of E14.5 neurospheres after an initial seeding of 3×10^5^ cells/ml derived from *Nbn^Ctrl^* (n = 21), *Nbn^Nes-Cre^* (n = 6) and *Nbn/Atm^Nes-Cre^* (n = 3) brains. (**C**) Numbers of cells in each neurospheres derived from *Nbn^Ctrl^* (n = 31), *Nbn^Nes-Cre^* (n = 10) and *Nbn/Atm^Nes-Cre^* (n = 5) brains. Error bars indicate SEM. (* p<0,05, ** p<0,01, *** p<0,001, ns: non-significant).

Since Nbn/Atm deficient mice exhibited premature growth defects and death as compared to Nbn-deficient ones, we investigated whether *Atm* inactivation could worsen the Nbn deficiency phenotype in the neural stem cells (NSCs). NSCs from *Nbn^Ctrl^, Nbn^Nes-Cre^* and *Nbn/Atm^Nes-Cre^* from E14.5 brains were isolated and cultured *in vitro* (Figure 2A). At this stage the deletion of both *Nbn* and *Atm* is completed using *Nestin-Cre* [15]. Both neurospheres derived from *Nbn^Nes-Cre^* and *Nbn/Atm^Nes-Cre^* brains exhibit substantial reduction in size and number (Figure 2B–C) as compared to those derived from control brains. Interestingly, no significant difference on the number of neurospheres per ml or number of cells per neurosphere was observed between NSCs derived from *Nbn^Nes-Cre^* or *Nbn/Atm^Nes-Cre^* mice (Figure 2B–C). These findings suggest that the slight differences arising later in life may occur during development due to the effects of Atm deficiency on specific cells.

### Atm prevents premature DSBs accumulation and apoptosis in Nbn-deficient cerebellum and forebrain

**Figure 3:**
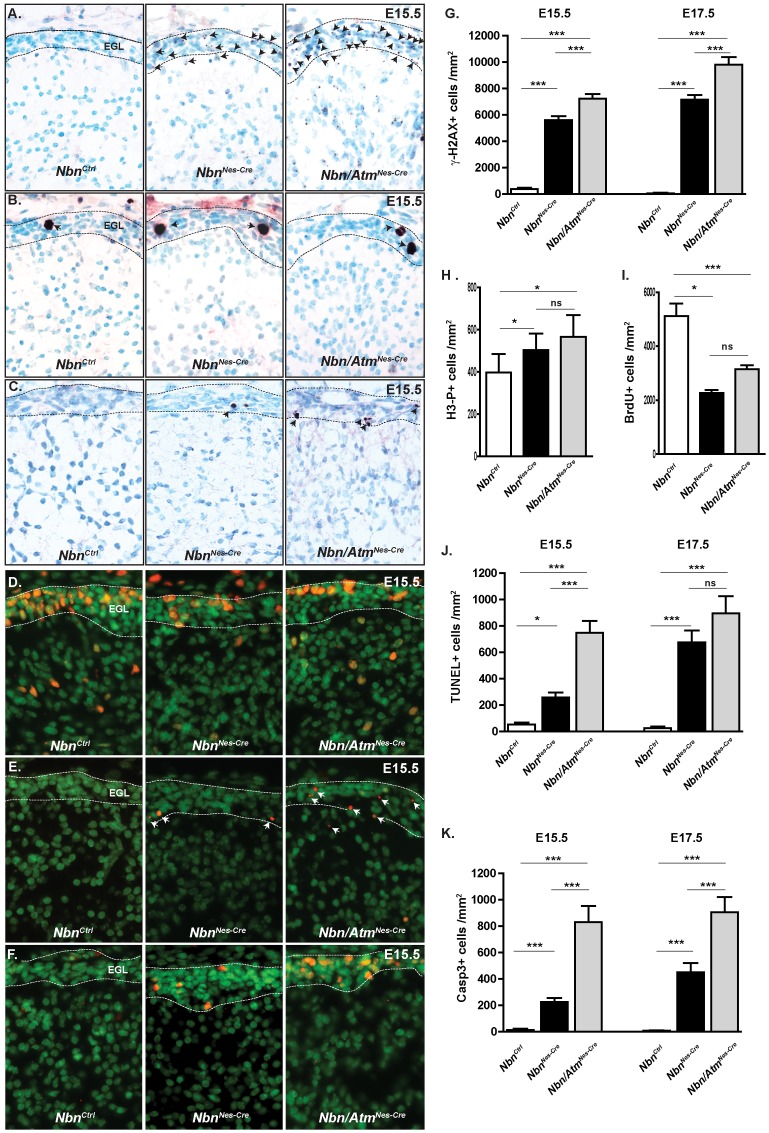
*Nbn* and *Atm* inactivation leads to accumulation of DSBs, proliferation defects and apoptosis in the EGL of the cerebellum. (**A**) Loss of Nbn and Atm leads to increase of DSBs at E15.5 as indicated by γ-H2AX foci (*Nbn^Ctrl^*, n = 3; *Nbn^Nes-Cre^*, n = 5 and *Nbn/Atm^Nes-Cre^*, n = 4) (Magnification ×400). (**B**) Accumulation of neural progenitors in mitosis in EGL primordium as indicated by H3 phosphorylation (Magnification ×400). (**C**) Cleaved Caspase-3 staining demonstrates increased apoptosis in *Nbn^Nes-Cre^* and *Nbn/Atm^Nes-Cre^* EGL. (Magnification ×400) (**D**) BrdU immunohistochemistry shows the reduction in cell proliferation of the neural progenitors in the *Nbn^Nes-Cre^* and *Nbn/Atm^Nes-Cre^* EGL (Magnification ×400). (**E**) TUNEL staining demonstrates increased apoptosis in *Nbn^Nes-Cre^* and *Nbn/Atm^Nes-Cre^* EGL (Magnification ×400). (**F**) p53 is stabilized in *Nbn^Nes-Cre^* and *Nbn/Atm^Nes-Cre^* EGL (Magnification ×400). (**G**) Quantification of cells with γ-H2AX foci in the EGL primordium at E15.5 (*Nbn^Ctrl^*, n = 3; *Nbn^Nes-Cre^*, n = 5 and *Nbn/Atm^Nes-Cre^*, n = 4) and E17.5 (*Nbn^Ctrl^*, n = 3; *Nbn^Nes-Cre^*, n = 3 and *Nbn/Atm^Nes-Cre^*, n = 3). (**H**) Quantification of phospho-histone 3 positive cells (H3-P+) in the EGL primordium at E15.5 (*Nbn^Ctrl^*, n = 4; *Nbn^Nes-Cr^* ^e^, n = 5 and *Nbn/Atm^Nes-Cre^*, n = 5). (**I**) Quantification of BrdU positive cells in the EGL primordium At E15.5 (*Nbn^Ctrl^*, n = 5; *Nbn^Nes-Cre^,* n = 3 and *Nbn/Atm^Nes-Cre^*, n = 4). (**J**) Quantification of TUNEL positive cells in the EGL primordium at E15.5 (*Nbn^Ctrl^*, n = 4; *Nbn^Nes-Cre^,* n = 3 and *Nbn/Atm^Nes-Cre^*, n = 4) and E17.5 ((*Nbn^Ctrl^*, n = 3; *Nbn^Nes-Cre^,* n = 3 and *Nbn/Atm^Nes-Cre^*, n = 4). (**K**) Quantification of Cleaved Caspase-3 positive cells in the EGL primordium at E15.5 (*Nbn^Ctrl^*, n = 3; *Nbn^Nes-Cre^,* n = 5 and *Nbn/Atm^Nes-Cre^*, n = 4) and E17.5 (*Nbn^Ctrl^*, n = 3; *Nbn^Nes-Cre^,* n = 3 and *Nbn/Atm^Nes-Cre^*, n = 4) Error bars indicate SEM. (* p<0,05, ** p<0,01, *** p<0,001, ns: non-significant).

To determine whether Atm deficiency would increase accumulation of DSBs in Nbn-deficient granule cells, we measured γ-H2AX foci in the cerebellum of E15.5 embryos. We found that the cells of the external germinal layer (EGL) of both *Nbn^Nes^* ^-*Cre*^ and *Nbn/Atm^Nes-Cre^* presented increased γ-H2AX foci compared to those of *Nbn^Ctrl^* (Figure 3A, 3G). Interestingly, the EGL from *Nbn/Atm^Nes-Cre^* exhibit significantly more cells with γ-H2AX compared to *Nbn^Nes-Cre^* granule cells, suggesting that Atm may play a role in the prevention of DSB accumulation in Nbn-deficient cells. DSBs are frequently associated with an activation of cell cycle checkpoints or apoptosis. Therefore, we first measured BrdU incorporation in EGL cells after a 1 hour BrdU pulse. Consistent with former studies, we found significant reduction of BrdU positive cells in EGL from Nbn-deficient cells. Simultaneous inactivation of *Atm* did not alter the proportion of BrdU positive cells (Figure 3D, 3I). In parallel, the number of cells in mitosis was evaluated using the phospho-histone 3 (phospho-H3) marker. A moderate increase in the number of phospho-H3 positive cells was found in *Nbn^Nes^* ^-*Cre*^ and *Nbn/Atm^Nes-Cre^* EGL (Figure 3B, 3H), suggesting that G2/M cell cycle checkpoint is activated in *Nbn^Nes^* ^-*Cre*^ and *Nbn/Atm^Nes-Cre^* deficient EGL cells. To test this hypothesis, we asked whether the DSBs increase would correlate with p53 protein stabilization. Indeed, in EGL of both *Nbn^Nes^* ^-*Cre*^ and *Nbn/Atm^Nes-Cre^* mice we found a high proportion of p53 positive cells (Figure 3F) with detectable phosphorylation at serine 18 (Figure S1). It indicates that in *Nbn^Nes^* ^-*Cre*^ and *Nbn/Atm^Nes-Cre^* EGL cells, p53 can be stabilized and phosphorylated at serine 18 independently of Nbn and Atm. Since p53 stabilization and apoptosis are often associated in the CNS, we analyzed apoptotic cell death using two independent markers: TUNEL and cleaved caspase-3. As expected, almost no TUNEL or cleaved caspase 3 positive cells were found in the control EGL at E15.5 (Figure 3C, 3K). In contrast, the proportion of apoptotic cells increased in both Nbn-deficient and Nbn/Atm-deficient cells (Figure 3E, 3J). Importantly, Nbn/Atm-deficient EGL exhibit a 4-fold increase of TUNEL and cleaved caspase 3 positive cells as compared to Nbn deficiency alone (p<0,001). Notably, in both genotypes, similar numbers of TUNEL positive cells and cleaved caspase 3 positive cells were observed, suggesting that at E15.5 the apoptosis is always caspase 3-dependent. At E17.5, we observed that the apoptosis in Nbn-deficient EGL cells reached the same level as in the Nbn/Atm-deficient EGL (Figure 3J, 3K). Here it is worth to notice that in *Nbn^Nes^* ^-*Cre*^ cerebella the apoptotic cells were localized in the external border of the EGL where most of the cells exit cell cycle, while in *Nbn/Atm^Nes-Cre^* cerebella, cells undergoing apoptotic cell death were localized throughout the EGL. It suggests that Atm is required to prevent apoptosis in dividing cells as well as in post-mitotic Nbn-deficient cells. In conclusion, Nbn deficiency in cerebellum primordium induces DNA DSBs, cell cycle checkpoint activation and stabilization of p53. Concomitant inactivation of *Atm* induces premature apoptosis, suggesting that Atm may be required to promote DNA repair and prevent apoptosis in Nbn-deficient damaged cells.

**Figure 4:**
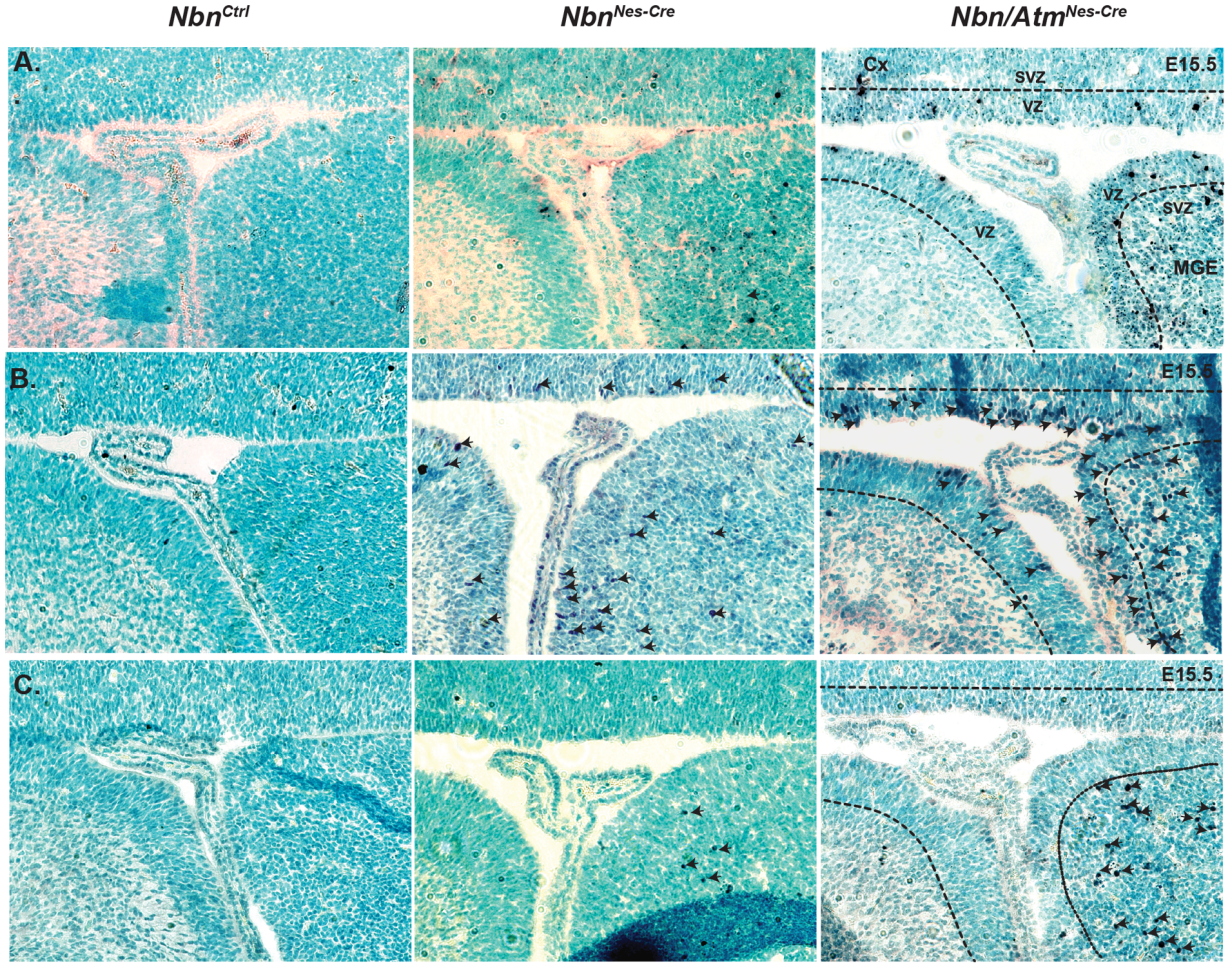
Inactivation of *Nbn* and *Atm* leads to increase of DSBs and apoptosis in medial ganglionic eminence (MGE). (**A**) Accumulation of γ-H2AX foci in MGE of *Nbn^Nes-Cre^* and *Nbn/Atm^Nes-Cre^* brains at E15.5 (Magnification ×200). (**B**) Immunohistochemistry indicates stabilization of p53 protein in the E15.5 MGE following *Nbn* and *Atm* inactivation (Magnification ×200). (**C**) Increased apoptosis in the SVZ from E15.5 MGE indicated by cleaved caspase-3 positive cells. Cx: Cortex, MGE: Medial Ganglionic Eminence, VZ:Ventricular Zone, SVZ:Sub ventricular Zone (Magnification ×200).

After investigating the consequences of *Nbn* and *Atm* genetic inactivation in cerebellum, we analyzed whether other areas of the CNS would be affected by Nbn or combined Nbn and Atm loss. We found that at E15.5 only the forebrain and in particular the medial ganglionic eminence (MGE) (source of the majority of interneurons in the neocortex) presented DSBs (γ-H2AX) (Figure 4A), DNA damage response, as verified by the stabilization of p53 protein (Figure 4B) and apoptosis (cleaved caspase 3) (Figure 4C). Interestingly, while γ-H2AX foci and p53 positive cells were found in ventricular zone (VZ) and sub-ventricular zone (SVZ) of the MGE (Figure 4A–B), apoptotic cells (positive for cleaved caspase-3) were localized only in the SVZ (Figure 4C). Similarly to what was observed in the cerebellum, *Atm* inactivation in Nbn-deficient neuronal cells increased DSBs, as indicated by γ-H2AX foci, p53 positive cells in the VZ and increased apoptosis in the SVZ (Figure 4). Later during the development (E17.5) only the VZ and SVZ of the neocortex exhibited this phenotype (data not shown). Altogether these data show similar consequences of simultaneous inactivation of *Nbn* and *Atm* in both cerebellum and forebrain. In addition, it suggests that the fate of progenitors presenting DSBs is intimately associated with their localization and most likely with the type of cell division.

### Atm inactivation exacerbates the growth impairment of Nbn-deficient lens due to DSBs accumulation and premature apoptosis

**Figure 5:**
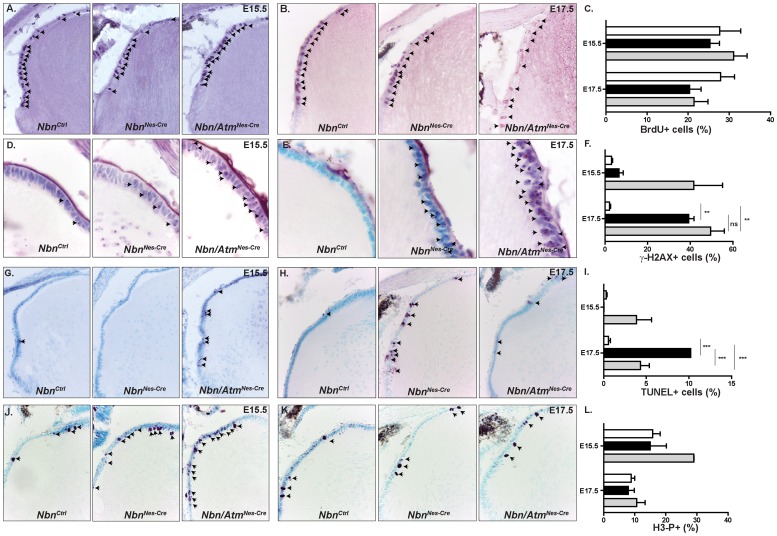
Inactivation of *Nbn* and *Atm* leads to precocious DSBs accumulation and apoptosis in embryonic lens. (**A**) BrdU immunohistochemistry of *Nbn^Ctrl^*, *Nbn^Nes-Cre^* and *Nbn/Atm* ^Nes-Cre^ lens at E15.5 (Magnification ×200). (**B**) BrdU immunohistochemistry of *Nbn^Ctrl^*, *Nbn^Nes-Cre^* and *Nbn/Atm* ^Nes-Cre^ lens at E17.5 (Magnification ×200). (**C**) Quantification of BrdU positive cells within lens anterior epithelia at E15.5 (*Nbn^Ctrl^*, n = 3; *Nbn^Nes-Cre^,* n = 3; and *Nbn/Atm^Nes-Cre^*, n = 3) and E17.5 (*Nbn^Ctrl^*, n = 7; *Nbn^Nes-Cre^,* n = 6; and *Nbn/Atm^Nes-Cre^*, n = 3). (**D**) γ-H2AX immunohistochemistry of *Nbn^Ctrl^*, *Nbn^Nes-Cre^* and *Nbn/Atm* ^Nes-Cre^ lens at E15.5 (Magnification ×1000). (**E**) γ-H2AX immunohistochemistry of *Nbn^Ctrl^*, *Nbn^Nes-Cre^* and *Nbn/Atm* ^Nes-Cre^ lens at E17.5 (Magnification ×1000). (**F**) Quantification of γ-H2AX positive cells within lens anterior epithelia at E15.5 (*Nbn^Ctrl^*, n = 3; *Nbn^Nes-Cre^,* n = 3 and *Nbn/Atm^Nes-Cre^*, n = 5) and E17.5 (*Nbn^Ctrl^*, n = 3; *Nbn^Nes-Cre^,* n = 3 and *Nbn/Atm^Nes-Cre^*, n = 3). (**G**) TUNEL staining of *Nbn^Ctrl^*, *Nbn^Nes-Cre^* and *Nbn/Atm* ^Nes-Cre^ lens at E15.5 (Magnification ×400). (**H**) TUNEL immunohistochemistry of *Nbn^Ctrl^*, *Nbn^Nes-Cre^* and *Nbn/Atm* ^Nes-Cre^ lens at E17.5 (Magnification ×400). (**I**) Quantification of TUNEL cells within lens anterior epithelia at E15.5 (*Nbn^Ctrl^*, n = 20; *Nbn^Nes-Cre^,* n = 2 and *Nbn/Atm^Nes-Cre^*, n = 5) and E17.5 (*Nbn^Ctrl^*, n = 7; *Nbn^Nes-Cre^,* n = 3 and *Nbn/Atm^Nes-Cre^*, n = 5). DSB accumulation and apoptotic cell death occurs earlier in Nbn/Atm-deficient lens as compared to Nbn-deficient lens. (**J**) Phospho-histone 3 (H3-P) immunohistochemistry of *Nbn^Ctrl^*, *Nbn^Nes-Cre^* and *Nbn/Atm* ^Nes-Cre^ lens at E15.5 (Magnification ×400). (**K**) Phospho-histone 3 (H3-P) immunohistochemistry of *Nbn^Ctrl^*, *Nbn^Nes-Cre^* and *Nbn/Atm* ^Nes-Cre^ lens at E17.5 (Magnification ×400). (**L**) Quantification of Phospho-histone 3 positive cells (H3-P+) cells within lens anterior epithelia at E15.5 (*Nbn^Ctrl^*, n = 6; *Nbn^Nes-Cre^,* n = 5 and *Nbn/Atm^Nes-Cre^*, n = 2) and E17.5 (*Nbn^Ctrl^*, n = 13; *Nbn^Nes-Cre^,* n = 3 and *Nbn/Atm^Nes-Cre^*, n = 5). Error bars indicate SEM. (* p<0,05, ** p<0,01, *** p<0,001, ns: non-significant).

Previous studies showed that Nbn was crucial for the development of both the retina and the lens [16], [19]. Nevertheless, the functional relationship of Nbn and Atm in these tissues has remained elusive. Therefore, we analyzed the development of the eye in both *Nbn^Nes^* ^-*Cre*^ and *Nbn/Atm^Nes-Cre^* mice. The *Nestin-Cre* transgenic mice present a high Cre recombinase activity in developing lens and retina [23]. First, we confirmed our previous reports that *in vivo* inactivation of *Nbn* impaired eye and lens growth (Figure 1I–L). At P9, the *Nbn^Ctrl^* eyes were bigger than Nbn-deficient ones (65.12±1.44 mm^3^ vs 51.07±1.92 mm^3^) and *Nbn/Atm^Nes-Cre^* eyes (41.77±1.02 mm^3^) were significantly smaller than *Nbn^Nes-Cre^* eyes (Figure 1I, 1K). Combined inactivation of *Nbn* and *Atm* also affected lens growth more severely than Nbn deficiency alone (*Nbn^Ctrl^* = 12.5±0.47; *Nbn^Nes-Cre^ = *7.98±0.50*; Nbn/Atm^Nes-Cre^* = 6.14±0.24) (Figure 1J–L). To understand how lens development was more severely affected by simultaneous inactivation of Nbn and Atm, we first quantified the number of proliferating cells using BrdU labeling. As previously shown, we observed a slight decrease in the number of BrdU positive cells in Nbn-deficient lens in late embryonic development (E17.5). The reduction in the proportion of BrdU positive cells was not altered by additional Atm loss (Figure 5A–C). Interestingly, the proportion of mitotic cells (phospho-H3 positive cells) was slightly increased only in Nbn/Atm-deficient lens, suggesting the activation of the G2/M cell cycle checkpoint (Figure 5J–L). To test whether Nbn and/or Atm loss would affect accumulation of DSBs and apoptosis in developing lens, we scored the proportion of γ-H2AX and TUNEL positive cells in *Nbn^Ctrl^, Nbn^Nes-Cre^* and *Nbn/Atm^Nes-Cre^* lens. At E15.5, a ∼10-fold increase in the proportion of γ-H2AX positive cells was observed exclusively in lens lacking both Nbn and Atm. At E17.5, the proportion of γ-H2AX positive was significantly increased (∼20-fold) in both *Nbn^Nes-Cre^* and *Nbn/Atm^Nes-Cre^* lens (Figure 5D–F). To verify whether the observed DSBs increase would affect cell survival during lens embryogenesis, we quantified the proportion of TUNEL positive cells in the lens epithelial cell layer. As expected, at E15.5, a 10-fold increase in apoptotic cells was exclusively detected in *Nbn/Atm^Nes-Cre^* lens, while at E17.5, Nbn-deficiency alone and combined loss of Nbn and Atm (∼20-fold) lead to apoptosis of lens progenitor cells (Figure 5G–I). To extend the results obtained with *Nestin-Cre*, we used a lens-specific mouse line (*Le-Cre*) [24] that allows deletion of the genes in early lens development, specifically in the surface ectoderm. We postulated that earlier deletion in lens progenitor cells population would augment the defects already observed in the *Nestin-cre*. As expected, at P9 the eye growth of *Nbn^Le-Cre^* and *Nbn/Atm^Le-Cre^* mice was already affected (data not shown) and at P21, the volume of the eyes were dramatically reduced (Figure S2A–B) in both *Nbn^Le-Cre^* and *Nbn/Atm^Le-Cre^* mice. Similarly to what was observed in *Nestin-Cre* mice, Atm deficiency potentiated the defects observed in Nbn-deficient lens (Figure S2A–B). Histological analysis revealed that at P21, the lens is severely reduced in the *Nbn^Le-Cre^* mice and completely absent in the *Nbn*/*Atm^Le-Cre^*. As expected, the severe impairment of lens development completely abrogated retinal development (Figure S2A–B).

Altogether, the analysis of cell proliferation, DNA damage and cell death indicate that both Nbn and Atm are crucial for lens progenitor cells survival during embryogenesis. Importantly, the more severe impairment of lens growth as a result of simultaneous inactivation of Nbn and Atm indicates that these proteins have independent roles in the developing eye.

### Nbn-deficiency leads to an Atm-dependent apoptosis in developing retina

**Figure 6:**
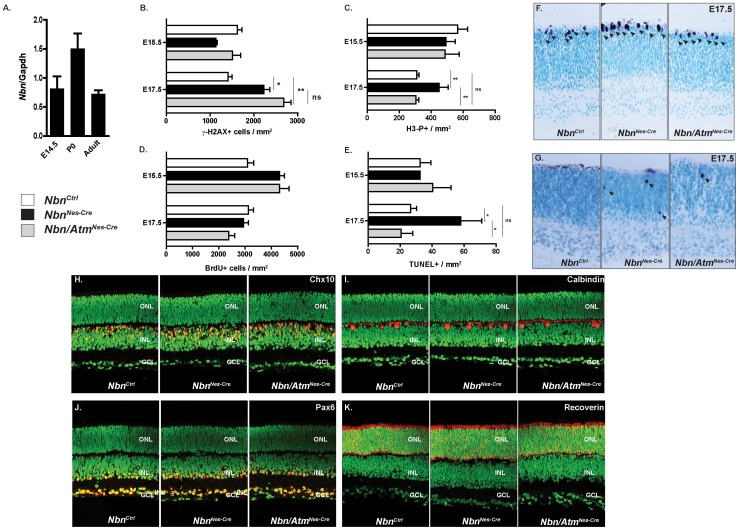
Nbn-deficiency leads to Atm-dependent apoptotic cell death in developing retina, but cell fate specification and differentiation are normal in the Nbn/Atm-deficient retina. (**A**) Relative expression of *Nbn* mRNA at different stages of mouse retinal development was analyzed by real time RT-PCR using SYBR green. No significant difference in the levels of *Nbn* mRNA expression was detected within the stages analyzed. Gene expression was normalized to Gapdh mRNA (*Gapdh*). (**B**) Quantification of γ-H2AX positive cells within retina at E15.5 (*Nbn^Ctrl^*, n = 3; *Nbn^Nes-Cre^,* n = 4; and *Nbn/Atm^Nes-Cre^*, n = 3) and E17.5 (*Nbn^Ctrl^*, n = 2; *Nbn^Nes-Cre^,* n = 3; and *Nbn/Atm^Nes-Cre^*, n = 3). (**C**) Quantification of H3-P positive cells per mm^2^ of retinal tissue at E15. 5 (*Nbn^Ctrl^*, n = 8; *Nbn^Nes-Cre^,* n = 6 and *Nbn/Atm^Nes-Cre^*, n = 5) and at E17.5 (*Nbn^Ctrl^*, n = 13; *Nbn^Nes-Cre^,* n = 3 and *Nbn/Atm^Nes-Cre^*, n = 6). (**D**) Quantification of BrdU positive cells within retina at E15.5 (*Nbn^Ctrl^*, n = 10; *Nbn^Nes-Cre^,* n = 6; and *Nbn/Atm^Nes-Cre^*, n = 12) and E17.5 (*Nbn^Ctrl^*, n = 8; *Nbn^Nes-Cre^,* n = 3; and *Nbn/Atm^Nes-Cre^*, n = 2). Error bars indicate SEM (* p<0,05, ** p<0,01, ns: non-significant). (**E**) Quantification of apoptotic cells indicated by TUNEL positive cells per mm^2^ of retinal tissue at E15.5 (*Nbn^Ctrl^*, n = 5; *Nbn^Nes-Cre^,* n = 1 and *Nbn/Atm^Nes-Cre^*, n = 4) and E17.5 (*Nbn^Ctrl^*, n = 15; *Nbn^Nes-Cre^,* n = 3 and *Nbn/Atm^Nes-Cre^*, n = 5) (**F**) Phospho-histone H3 (H3-P) immunohistochemistry of *Nbn^Ctrl^*, *Nbn^Nes-Cre^* and *Nbn/Atm* ^Nes-Cre^ retina at E17.5 (Magnification ×400). (**G**) TUNEL staining of *Nbn^Ctrl^*, *Nbn^Nes-Cre^* and *Nbn/Atm* ^Nes-Cre^ retina at E17.5 (Magnification ×400). (**H–K**) Retinal cryosections of P9 retinas from *Nbn^Ctrl^*, *Nbn^Nes-Cre^* and *Nbn/Atm^Nes-Cre^* were stained with cell type–specific antibodies as shown, in red, for Chx10 (bipolar cells) (**H**), for calbindin (horizontal cells) (**I**), for Pax6 (amacrine cells) (**J**) and for recoverin (photoreceptors and a subset of bipolar cells) (**K**). Nuclei are shown in green (sytox green counterstaining). Immunohistochemistry patterns of all cell types analyzed are indistinguishable between control, Nbn-deficient and Nbn/Atm-double-deficient retinas (Magnification ×400). Abbreviations: ONL, outer nuclear layer; INL, inner nuclear layer; GCL, ganglion cell layer. Error bars indicate SEM. (* p<0,05, ** p<0,01, ns: non-significant).

Previous findings demonstrated that Nbn loss compromises the development of the visual system and indicated that Nbn-deficiency results in neurodegeneration in adult retina [16]. Here, we asked whether Nbn and Atm regulates early stages of retinal development. Initially, we performed real time PCR to analyze the expression of *Nbn* in various stages of retinal development. In the mouse retina, Nbn is expressed as early as E14.5 (Figure 6A). To test whether Nbn and/or Atm would regulate genome stability in retinal progenitor cells, we quantified γ-H2AX positive cells in *Nbn^Ctrl^, Nbn^Nes-Cre^* and *Nbn/Atm^Nes-Cre^* embryonic retinas. At E17.5, a slight increase in the proportion of γ-H2AX positive cells was observed in both Nbn- and Nbn/Atm-deficient retinas (Figure 6B). To verify whether the detected DSBs would affect cell cycle progression, we first quantified the proportion of BrdU positive cells. Nbn-deficiency or Nbn/Atm-deficiency did not significantly altered BrdU incorporation in E15.5 or E17.5 retinas (Figure 6D). To directly analyze the mitotic subpopulation of retinal progenitors, we scored the proportion of phospho-H3 positive cells. At E17.5, *Nbn* inactivation increased the proportion of mitotic cells. Surprisingly, in contrast to what was observed in the lens, this effect was completely reverted by *Atm* inactivation. These findings suggest that Nbn-deficiency induced an Atm-dependent G2/M arrest of the cell cycle (Figure 6C, 6F). To provide additional evidence of Nbn role in retinal development, we asked whether its inactivation would regulate cell death in embryonic retina. At E17.5, Nbn loss induced a 2-fold increase in the number of TUNEL positive cells in the neuroblastic layer (NBL) of the retina (Figure 6E, 6G). As already observed for phospho-H3 positive cells accumulation, apoptosis induced by Nbn-deficiency, was completely blocked by inactivation of *Atm*, suggesting that in developing retina both the G2/M arrest and the apoptotic cell death are mediated by an Atm-dependent pathway.

### Nbn and Atm are not required for neuronal differentiation during retinal development

To test whether retinal cell fate specification and differentiation would be compromised in the absence of Nbn and/or Atm function, we analyzed the distribution of various retinal cell types in *Nbn^Ctrl^*, *Nbn^Nes-Cre^* and *Nbn/Atm^Nes-Cre^* postnatal retinas. Bipolar cells (Chx10, Figure 6H), horizontal cells (calbindin, Figure 6I), amacrine cells (Pax6, Figure 6J) and photoreceptor cells (recoverin, Figure 6K) were normally generated and distributed in Nbn-deficient or Nbn/Atm-deficient P9 retinas. Similar results were obtained when *Nbn* and *Atm* were inactivated at earlier stages of retinal development by the use of *Pax6-Cre* mice line [25]. At P28, no difference in the lamination or distribution of the analyzed retinal cell types was observed when *Nbn^Ctrl^*, *Nbn^Pax6-Cre^* and *Nbn/Atm^Pax6-Cre^* were compared (Figure S2C–E). These findings indicate that, in the mouse retina, the programs of neuronal and glial differentiation are not disturbed by genetic inactivation of *Nbn* and *Atm*.

## Discussion

In this study, we analyzed how Nbn and Atm cooperate *in vivo* to prevent DSBs accumulation and apoptosis during CNS and eye development. Understanding the functional interaction between Nbn and Atm *in vivo* is of prime importance considering that human neurological diseases such as NBS and A-T are associated with mutations in *NBN* and *ATM*. We provide evidence that both Nbn and Atm play important roles in the maintenance of genomic integrity in different areas of the developing CNS and in the eye. More importantly, we showed that the functional cooperation between Nbn and Atm is both tissue- and developmental stage-specific. Additionally, our findings support the idea that the neurological malformations observed in Nbn and Nbn/Atm-deficient mice are developmental, rather than resulting from neurodegeneration.

We expected that combined inactivation of *Nbn* and *Atm* would augment the developmental defects associated with *Nbn* deletion during CNS development because of the synthetic lethality already observed in *Nbn* ^ΔB/ΔB^, *Atm* ^−/−^ mice [14] or in the acute developmental defects of the *Nbn*-CNS-Δ/ *Atm* ^−/−^ cerebella [17]. We demonstrate that instead of broadly compromising the entire neurogenesis, inactivation of *Nbn* or *Nbn*/*Atm* affected only few areas of the developing CNS and eye, including the cerebellum, the GE and the lens, as well as the retina and did not compromise survival of differentiated neurons. The cerebellum and in particular the granule cells (GCs) were already shown to be highly sensitive to *Nbn* inactivation [15], [17], [26], [27]. The sensitivity of cerebellum to DSBs genes deficiency was often attributed to the extremely fast cell proliferation of the GCs at a certain stages of development, which would render them more susceptible to apoptosis associated with replication forks collapse [28]. Besides, increased apoptosis during EGL development in Nbn- and Nbn/Atm-deficient cerebella was untimely correlated with the increased proliferation of GCs progenitors. Furthermore, we found that the viability of differentiated neurons from the cerebellum, such as Purkinje cells did not require Nbn or Atm and that these cells did not present spontaneous DSBs. Despite numerous studies, the function of the MRN complex in differentiated neurons remains controversial. Indeed, a marked reduction of Purkinje cells in *Nbn*-CNS-Δ [26] and *Nbn*-CNS-Δ/ *Atm* ^−/−^ [17] was reported. In contrast, consistent with the data presented in this study, no defects were observed in Purkinje cells following inactivation of *Nbn* [27] or *Rad50* [29] using *Pcp2-cre* even in a long term study. Several other findings support the hypothesis that Nbn and Atm are dispensable for the homeostasis of differentiated Purkinje cells. Purkinje cells in mouse exit the cell cycle early during cerebellar development around E12.5 making them less sensitive to the DNA damage associated with long term proliferation and replicative stress [30]. Moreover, the remaining GCs may be sufficient to maintain Purkinje cells viability despite their altered branching as observed in other mouse models [31], [32]. It was shown that post-mitotic and differentiated neurons repair DSBs resulting from oxidative stress mainly by Non Homologous End Joining (NHEJ) [33]. In addition, Nbn has been shown to be dispensable for classic NHEJ and *Nbn* inactivation was shown to favor NHEJ in mouse cells [9], [34], [35]. In conclusion, similarly to what was shown for Rad50, we demonstrated that Nbn is dispensable for the viability of post-mitotic differentiated neural cells.

In our study, we show also that in addition to the cerebellum, the SVZ from the developing forebrain composed of four regions the medial ganglionic eminence (MGE), the lateral ganglionic eminence the caudal ganglionic eminence and the fetal neocortical SVZ presented an acute sensitivity to both Nbn and Nbn/Atm deficiency (Figure 4). Among them the MGE appeared to be the most affected with severe apoptosis as early as E15.5. The MGE has been shown to give rise to a tremendous variety of cell types populating various CNS structures including cortex, striatum, thalamus, hippocampus and olfactory bulbs [36], [37]. It is the major source of the GABAergic interneurons of the cortex populating its layers II to V, and representing up to 30% of its neural population [38]. The MGE was also demonstrated to be a source of oligodendrocytes [39]. In human, the lack of cortex interneurons or their defective migration and localization were often associated with diseases such as autism, epilepsy or seizures [40]. This is consistent with some NBS patients suffering severe epilepsy [41] and other presenting various form of neuronal migration disorder including schizencephaly [42], pachygyria [43] and partial lissencephaly [41]. In addition *Nbn* ^CNS-del^ mice exhibit severe disruption of olfactory bulbs morphology and reduced cellularity of hippocampus and of the layers II to V from the cortex [44]. Altogether, we demonstrate that Nbn is essential for the cell survival in ganglionic eminences and the formation of interneurons. We also propose a cause for the cortical defects observed in both in NBS patients [41] and *Nbn*-deficient mice [44].

We also found that the eye development was severely affected by Nbn and Atm deficiencies. These findings resemble the human syndromes Ataxia-Telangiectasia (A-T), and Nijmegen Breakage Syndrome (NBS), which often exhibit developmental or neurodegenerative defects in ocular structures. Ataxia-Telangiectasia (A-T) patients present conjunctival/scleral telangiectasia [45], [46], saccadic abnormalities [47], head thrusts, strabismus and abnormal convergence [48]. Surprisingly, the A-TLD syndrome that results from MRE11 mutations and shares close similarities with A-T, exhibits moderated eye defects [48]. NBS patients exhibit also some ophthalmological abnormalities including reduced visual acuity, smaller eyes, cornea diameter lens opacity and refractive errors [49]. In mice, previous studies demonstrated that Nbn function was required for proper lens development. Yang and colleagues concluded that cell proliferation and altered differentiation were the causes of the defects in Nbn-deficient lens [19]. Complementary analysis performed here suggested that cell cycle arrest and apoptotic cell death are associated with the lens defects resulting from increased DSBs within lens progenitor cells. Indeed, we already detected increased γ-H2AX foci in both Nbn- and Nbn/Atm-deficient lens epithelial cells as early as E15.5. Both the increased DNA damage in proliferating cells and the apoptotic cell death started earlier in Nbn/Atm-deficient, indicating that Atm may promote DSBs repair in Nbn-deficient lens progenitor cells and that additional deletion of Atm may lead to broader effects on both dividing and differentiating cells in Nbn-deficient lens. Additionally, we provide evidence that Nbn and Atm are required at very early stages in lens development since inactivation of *Nbn* in the surface ectoderm (using *Le-Cre* mice) dramatically impaired eye growth and lens development was completely abrogated in *Nbn/Atm^Le-Cre^* mice.

Surprisingly, in contrast to the lens, the embryonic retinal development was only mildly affected by *Nbn* inactivation. Our findings add to previous reports that showed decreased electroretinogram amplitudes, demyelination of optic nerve, axonal degeneration in addition to oligodendrocytes and ganglion cells degeneration in adult Nbn-deficient eyes [16]. Indeed, our study indicates that Nbn regulates genomic integrity in retinal progenitor cells (RPC) during embryonic development. Inactivation of Nbn increases the number of RPC with DSBs indicating that, in the absence of MRN complex, DNA damage repair in RPC does not occur properly. Interestingly, the increased proportion of RPC containing DSBs observed in *Nbn*-deficient retina was not significantly altered by simultaneous Atm inactivation. In contrast, apoptosis associated with Nbn deletion was significantly decreased following Atm loss. This is in agreement with the requirement of Atm for apoptosis in developing retina upon irradiation [50]. Persistence of phosphorylation of H2AX in Nbn/Atm-deficient retina strongly suggest that at least one alternative kinase, is able to phosphorylate H2AX independently of both Nbn and Atm in RPC. It has been proposed that DNA-PKcs may regulate retinal development [51]. In addition, after analyzing the distribution of all retinal cell types right after the vast majority of RPC have become post-mitotic (P9), we provide evidence that cell fate specification or early steps of cell differentiation of retinal neurons and glia are not affected by Nbn and/or Atm deficiency. Therefore, we propose that the visual abnormalities described for mice with Nbn-deficient retina [16] most likely result from neuronal and/or glial degeneration during adulthood, but not from developmental malformations.

The similarity of the cellular defects and their timing of occurrence in the tissues affected by Nbn deficiency imply the existence a common molecular mechanism. We hypothesize that complete disruption of Nbn leads to replication fork collapses and the formation of DSBs as indicated by γ-H2AX foci in dividing cells. This is consistent with the role of Nbn in preventing the formation of DSBs by favoring stabilization and restart of stalled replication forks directly through activation and retention of ATR at the damage sites [52] or interaction with TopBP1 an ATR activator [53]. Notably, the phosphorylation of H2AX at Ser139 requires neither Nbn nor Atm since both Nbn and Nbn/Atm deficient cells presented foci. We suggest that ATR may be the kinase responsible for γ-H2AX, since Nbn has been shown to be dispensable for ATR mediated H2AX phosphorylation [52]. Nevertheless, it is not possible to exclude DNA-PKcs since it may compensate Atm loss in an efficient way and it was also shown to play a role in the survival of neural cells [51], [54]–[56]. In dividing cells, during the first part of the embryonic development, the DSBs trigger a survival signal, through the activation of cell cycle checkpoint mediated by p53 and others. p53 was already shown to be one of the key effectors to activate cell cycle checkpoint and apoptosis in the CNS [1]. The essential function of p53 was also highlighted by the partial or complete rescue of the neurological defects in several DSBs mouse mutants [28]. Interestingly, in cerebellum, the stabilization and phosphorylation at serine 18 of p53 is independent of both Nbn and Atm in contrast to what happens upon low dose of ionizing radiation. In this regard, the cerebellum and lens may slightly differ since p53 has been shown not to rescue the lens phenotype in Nbn-deficient eye [19]. The increase of apoptosis in Nbn/Atm versus Nbn-deficient cells in early embryonic development can be attributed to the essential requirement of Atm in the repair of DSBs resulting from replication forks collapses and activation of Rad51-dependent HR [57] and also the essential function of Atm in the DNA damage response in post-mitotic cells [58]. In late embryonic development the acceleration of cell division, the DSBs repair switch from canonical NHEJ to micro homology-mediated end joining (MMEJ) which requires the MRN complex [59], [60] may favor accumulation of unrepaired DSBs to an unsustainable level for the cells leading irremediably to a p53-dependent apoptosis. Indeed, It is known that the accumulation of unrepaired persistent lesions leads irremediably to apoptosis in the CNS [28], and that neural cells presented a gradient of tolerance to DSBs inversely correlate with their differentiation status [61].

To conclude, our study reveals new findings about the possible origins of ocular and cortical developmental defects of NBS patients. Besides, we demonstrated tissue-specific and developmental stage-specific requirements of Nbn and Atm acting synergistically to prevent accumulation of DSBs and apoptosis in CNS and eye.

## Materials and Methods

### Ethics statement

All animal care and procedures followed German legal regulations and were previously approved by the governmental review board of the state of Baden-Württemberg (Regierungspräsidium Karlsruhe-Abteilung 3-Landwirtschaft, Ländlicher Raum, Veterinär-und Lebensmittelwesen, Germany, licence G-114/11, experimental projects DKFZ 220, 241, 256). All the aspects of the mouse work were carried out following strict guidelines to insure careful, consistent and ethical handling of mice.

### Mice

The *Nbn* floxed and *Atm* floxed mice were as described [15], [31], [62]. *Nestin-Cre* (B6.Cg(SJL)-Tg(Nes-cre)1Kln/J) and *PcP2-Cre* (B6.129-Tg(Pcp2-cre)2Mpin/J) mice were purchased from the Jackson Laboratory. *Lens-Cre* (FVB-Tg(Pax6-cre, GFP)1Pgr/PgrCnrm, EM00755) and *ECG*/*Pax6-Cre* (FVB-Tg(Pax6-cre, GFP)2Pgr/PgrCnrm, EM00756) were obtained from EMMA. The control group (*Nbn^Ctrl^*) depending of the mouse Cre line was composed of *Nbn* ^+/+^, *Nbn* ^+/F^;*Nbn* ^F/F^;*Nbn* ^+/F^, *Atm* ^+/F^;*Nbn* ^F/F^, *Atm* ^+/F^;*Nbn* ^+/F^, *Atm* ^F/F^; *Nbn* ^+/+^, Cre^+/−^; *Nbn* ^+/F^, Cre^+/−^; *Nbn* ^+/F^, *Atm* ^+/F^, Cre^+/−^; *Nbn* ^+/F^, *Atm* ^F/F^, Cre^+/−^. Mice of other experimental groups were identified as follows: *Nbn^Nes-Cre^* =  *Nbn* ^F/F^, *Nestin-Cre* ^+/−^; *Atm^Nes-Cre^* = *Atm* ^F/F^, *Nestin-Cre* ^+/−^, *Nbn/Atm^Nes-Cre^* = *Nbn* ^/F^, *Atm* ^F/F^, *Nestin-Cre* ^+/−^; *Nbn^PcP2-Cre^* = *Nbn* ^F/F^, *PcP2-Cre* ^+/−^; *Atm^PcP2-Cre^* = *Atm* ^F/F^, *PcP2-Cre* ^+/−^; *Nbn/Atm^PcP2-Cre^* = *Nbn* ^F/F^, *Atm* ^F/F^, *PcP2-Cre* ^+/−^; *Nbn^Le-Cre^* = *Nbn* ^F/F^, *Lens-Cre* ^+/−^; *Atm^Le-Cre^* = *Atm* ^F/F^, *Lens-Cre* ^+/−^; *Nbn/Atm^Le-Cre^*: *Nbn* ^F/F^, *Atm* ^F/F^, *Lens-Cre* ^+/−^; *Nbn^Pax6-Cre^* = *Nbn* ^F/F^, *Pax6-Cre* ^+/−^; *Atm^Pax6-Cre^* = *Atm* ^F/F^, *Pax6-Cre* ^+/−^; *Nbn/Atm^Pax6-Cre^* = *Nbn* ^F/F^, *Atm* ^F/F^, *Pax6-Cre* ^+/−^.

### RNA extraction, cDNA synthesis, and real-time RT-PCR analysis

Retinal tissue was obtained from staged embryonic (E14.5, E17.5) and postnatal (P0, P3, P11, adult) C57BL/6 mice. RNA extraction and cDNA synthesis were performed as previously described [63], [64]. *Nbn* primers used were as follows: forward- 5′- ACCCACCCATTGATGAACCAGCTA-3′ and reverse 5′-CTGAGTTTCTTGTGCTGCTTGGCA-3′. Real time RT-PCR reactions were done with Power SYBR Green PCR Master Mix and in ABI7500 machine (Applied Biosystems). PCR product identity was verified by electrophoresis and by melting-point analysis. *Nbn* gene (Nbn) expression was normalized to Gapdh (Gapd) [65] and gapdh-normalized amount of *Nbn* gene expression was plotted relative to the mean of delta Ct of all ages analyzed.

### Histology and Immunohistochemistry

Embryos, eyes and brains were collected in 4% (w/v) phosphate-buffered saline (PBS)-buffered paraformaldhehyde (PFA) for 24 hours at 4 °C and cryoprotected overnight in 25% PBS-buffered sucrose (w/v) solution. Embryos, eyes and brains were sectioned at 10 μm using a Leica CM1950 cryostat. An antigen retrieval with citrate buffer was performed prior the antibodies incubation. Immunoreactivity was visualized using ABC complex and VIP substrate Kit or (Vector labs). The following antibodies were used:anti-Pax6 (mouse, 1/20, DSHB), anti-recoverin (rabbit, 1/2000, Chemicon), anti-glutamine synthetase (mouse, 1/1000, BD Biosciences), anti-Chx10 (sheep, 1/1000, ExalphaBiologicals), anti-Calbindin (mouse, 1/2000, Sigma), anti-PCNA (mouse, 1/500. Santa Cruz Biotechnology), Ki67 (rabbit, 1/1000, Thermo-Fisher scientific), anti-BrdU (rat, 1/100, abcam), Anti-active caspase-3 (rabbit, 1/100, BD Biosciences), anti-p53 (CM5, 1/500, Novocastra), anti-Ser15 p53 (rabbit, 1/100, Cell signaling), Anti-Ser139 γH2AX (mouse, 1/1000, Millipore), Anti-Ser139 γH2AX (rabbit, 1/500, Abcam), anti-Ser10 pH3 (rabbit, 1/500, Cell Signaling), anti-Ser10 pH3 (mouse, 1/500 Cell signaling). Immunofluorescence reactions for the retina and lens studies were developed with Cy3-tyramide (Perkin Elmer) and nuclear staining done with Sytox Green (1/15000, Invitrogen). For the *in vivo* proliferation study, mice were injected intraperitonally with 50 μg / g body weight with BrdU (Sigma-Aldrich). Embryos, eyes and brains were collected 1 hour after injection. TUNEL analysis was performed as described [66]. Images were captured with a Leica TCS-SP5 with an AOBS system.

### Morphometric and Volume measurements of the eye and lens

Eyes from embryonic, newborn (P9) and adult mice (P30) were processed and measured as previously described [63]. After enucleation, eyes were fixed in 4% phosphate-buffered paraformaldehyde. The axial length and two coronal axes (dorso-ventral and medial-lateral) of each eye were measured with a digital paquimeter and the eye volume was calculated after applying formula (4/3*PI)*x*y*z. After dissection of the retina and lens, the same procedure was used to calculate lens volume.

### Neurosphere culture

Neurosphere cultures were isolated and cultured as described [15], [32].

### Statistical analysis

Quantification analysis of cell cycle and cell death, immunopositive cells in the EGL, lens and retina were counted in at least 4 representative sections and mean values were calculated and statistically analyzed. These data were collected from at least 3 different animals per each group. One way ANOVA followed by post hoc Newman-Keuls or T-Test analysis were performed using Prism (v5.0, Graphpad). Differences were considered significant when P value was 0.05.

## Supporting Information

Figure S1 ***Nbn*** **and** ***Atm*** **inactivation leads to p53 phosphorylation at serine 18 in the EGL of the cerebellum.** p53 stabilization is associated with its phosphorylation at serine 18 in *Nbn^Nes-Cre^* and *Nbn/Atm^Nes-Cre^* EGL (Magnification ×400).
(TIF)

Figure S2 **Inactivation of** ***Nbn*** **and** ***Atm*** **in the surface ectoderm severely impairs eye development, but their specific inactivation in retina progenitor cells does not affect eye growth or retinal cell differentiation.** (**A**) Immunohistochemistry for calbindin (horizontal cells) and for recoverin (photoreceptors and a subset of bipolar cells). (**B**) in cryosections of adult (P28) retinas from *Nbn^Ctrl^*, *Nbn^Pax6-Cre^* and *Nbn/Atm^Pax6-Cre^* mice. Cell type–specific stainings are shown in red and nuclei are shown in green (Magnification ×400). Inactivation of *Nbn* and *Atm* in early stages of retinal embryonic development did not impair retinal cell fate specification or the differentiation of retinal neurons and (**C**) slightly reduced of eye volume in the *Nbn/Atm^Pax6-Cre^* mice. *Nbn^Ctrl^* (n = 7), *Nbn^ Pax6-Cre^* (n = 5) and *Nbn/Atm^ Pax6-Cre^* (n = 5). (**D**) Severe eye developmental defects as a consequence of inactivation of *Nbn* (*Nbn^Le-Cre^*) or both *Nbn* and *Atm* (*Nbn/Atm^Le-Cre^*) in the surface ectoderm (SE) (**E**) Severe impairment of eye growth associated with deletion of *Nbn* and *Atm* specifically in the SE. *Nbn^Ctrl^* (n = 10), *Nbn^Le-Cre^* (n = 4) and *Nbn/Atm^Le-Cre^* (n = 8). Error bars indicate SEM (* p<0,05, *** p<0,001, ns: non-significant). Abbreviations: ONL, outer nuclear layer; INL, inner nuclear layer; GCL, ganglion cell layer.
(TIF)
