# Correction: Nbn and Atm Cooperate in a Tissue and Developmental Stage-Specific Manner to Prevent Double Strand Breaks and Apoptosis in Developing Brain and Eye

**DOI:** 10.1371/annotation/b38f50e0-04b6-42c5-8b19-50be747a38f3

**Published:** 2013-08-22

**Authors:** Paulo M. G. Rodrigues, Paulius Grigaravicius, Martina Remus, Gabriel R. Cavalheiro, Anielle L. Gomes, Maurício Rocha-Martins, Lucien Frappart, David Reuss, Peter J. McKinnon, Andreas von Deimling, Rodrigo A. P. Martins, Pierre-Olivier Frappart

The correct name of the sixth author is Maurício Rocha-Martins.

