# Supplementary figures and images for: Nbn and Atm Cooperate in a Tissue and Developmental Stage-Specific Manner to Prevent Double Strand Breaks and Apoptosis in Developing Brain and Eye
Source: PLoS One. 2013 Jul 30;8(7):e69209. doi: 10.1371/journal.pone.0069209 (PMC3728324; doi:10.1371/journal.pone.0069209)

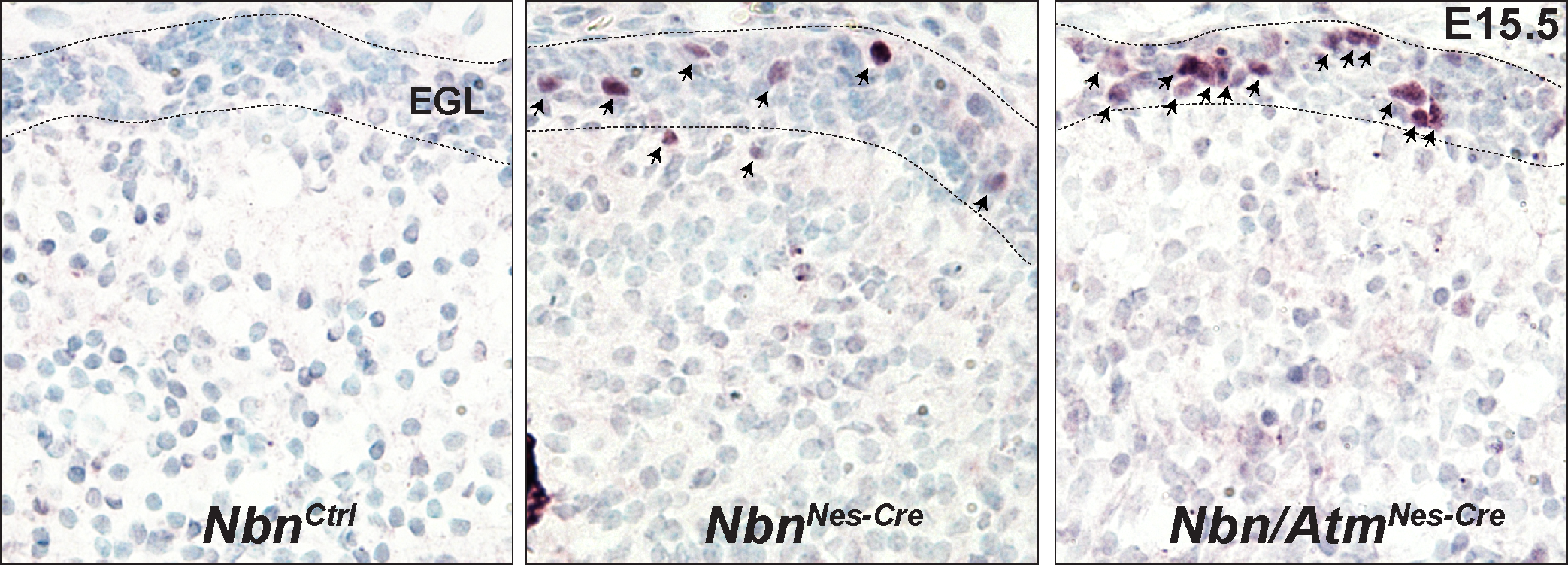

Supplement: Figure S1 — Nbn and Atm inactivation leads to p53 phosphorylation at serine 18 in the EGL of the cerebellum. p53 stabilization is associated with its phosphorylation at serine 18 in NbnNes-Cre and Nbn/AtmNes-Cre EGL (Magnification ×400). (TIF) [file pone.0069209.s001.tif]

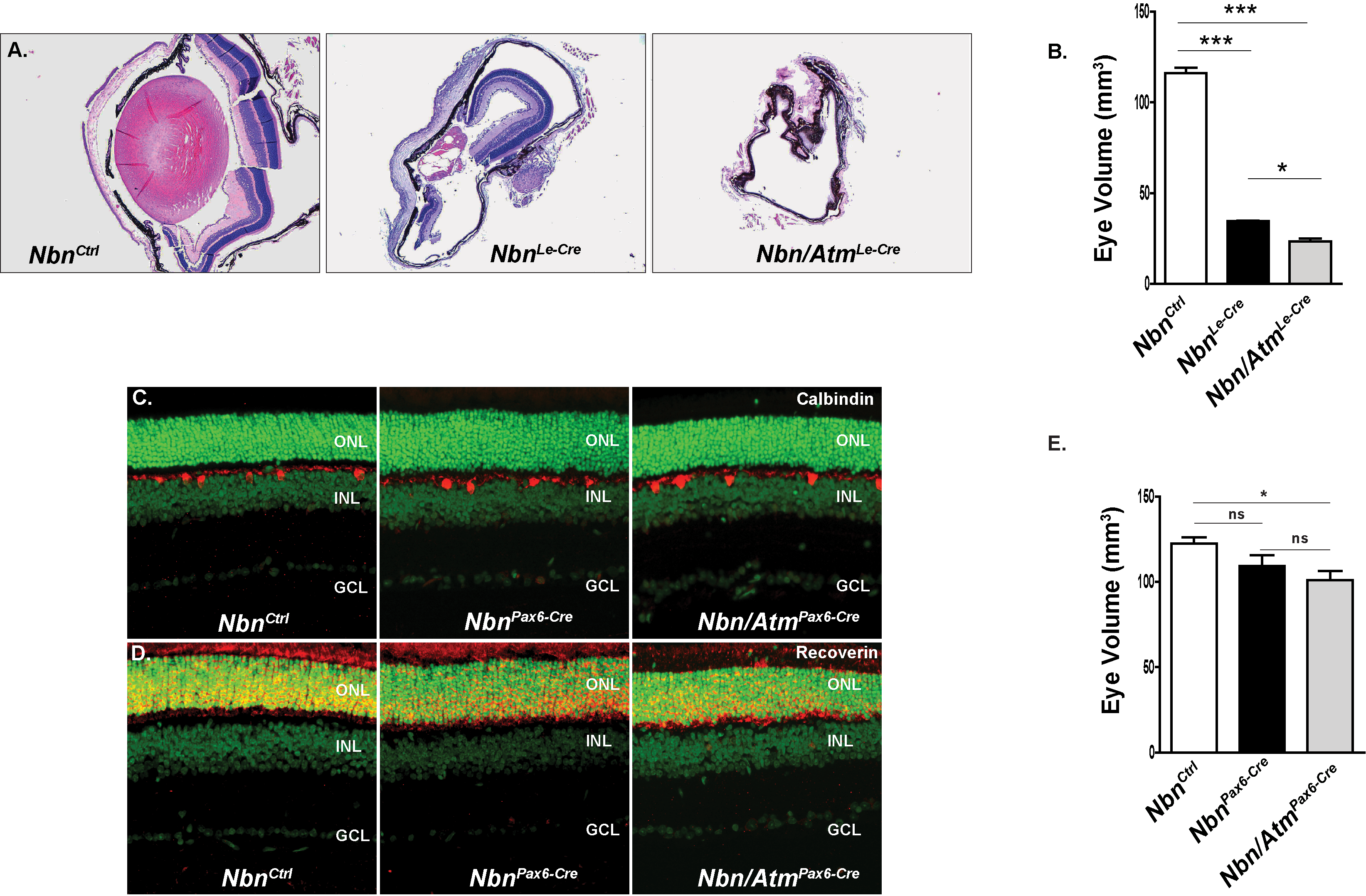

Supplement: Figure S2 — Inactivation of Nbn and Atm in the surface ectoderm severely impairs eye development, but their specific inactivation in retina progenitor cells does not affect eye growth or retinal cell differentiation. (A) Immunohistochemistry for calbindin (horizontal cells) and for recoverin (photoreceptors and a subset of bipolar cells). (B) in cryosections of adult (P28) retinas from NbnCtrl, NbnPax6-Cre and Nbn/AtmPax6-Cre mice. Cell type–specific stainings are shown in red and nuclei are shown in green (Magnification ×400). Inactivation of Nbn and Atm in early stages of retinal embryonic development did not impair retinal cell fate specification or the differentiation of retinal neurons and (C) slightly reduced of eye volume in the Nbn/AtmPax6-Cre mice. NbnCtrl (n = 7), Nbn Pax6-Cre (n = 5) and Nbn/Atm Pax6-Cre (n = 5). (D) Severe eye developmental defects as a consequence of inactivation of Nbn (NbnLe-Cre) or both Nbn and Atm (Nbn/AtmLe-Cre) in the surface ectoderm (SE) (E) Severe impairment of eye growth associated with deletion of Nbn and Atm specifically in the SE. NbnCtrl (n = 10), NbnLe-Cre (n = 4) and Nbn/AtmLe-Cre (n = 8). Error bars indicate SEM (* p<0,05, *** p<0,001, ns: non-significant). Abbreviations: ONL, outer nuclear layer; INL, inner nuclear layer; GCL, ganglion cell layer. (TIF) [file pone.0069209.s002.tif]
